# Print media coverage of primary healthcare and related research evidence in South Africa

**DOI:** 10.1186/s12961-015-0051-6

**Published:** 2015-11-12

**Authors:** Olagoke Akintola, John N Lavis, Ryan Hoskins

**Affiliations:** School of Applied Human Sciences, University of KwaZulu-Natal, Mazisi Kunene Road, Glenwood, Durban, 4041 South Africa; School of Human and Social Development, Nipissing University, 100 College Drive, ON P1B 8 L7 North Bay, Canada; McMaster Health Forum, McMaster University, 1280 Main St. West, MML 417, Hamilton, ON L8S 4 L6 Canada; Department of Clinical Epidemiology and Biostatistics, McMaster University, 1280 Main St. West, Hamilton, ON L8S 4 K1 Canada; Centre for Health Economics and Policy Analysis, McMaster University, CRL 209, 1280 Main St. West, Hamilton, ON L8S 4 K1 Canada; Department of Political Science, McMaster University, 1280 Main St. West, Hamilton, ON Canada; Department of Global Health and Population, Harvard School of Public Health, Boston, USA; Alberta Health Services, 1701-10010 119 St., Edmonton, Alberta T5K1Y8 Canada

**Keywords:** Community health worker, Community-based care, Health policy, Home-based care, Media analysis, Newspaper, Primary healthcare, Research evidence

## Abstract

**Background:**

The news media is located at the nexus of the public and policy agendas and provides a window into issues concerning the public. Therefore, it could be a powerful tool for advocating for citizens’ health and could help promote evidence-based primary health systems responsive to the needs of citizens. However, research on the coverage of primary healthcare and related research evidence in the South African print media is virtually non-existent.

**Methods:**

We examined 2,077 news stories that covered primary healthcare from 25 South African newspapers retrieved from the Lexis-Nexis online archive over a 16-year period (1997–2012). We analysed basic characteristics and conducted a content analysis of the news stories.

**Results:**

Of the 2,077 news stories that mentioned primary healthcare, this was the main focus in 8.3% (n = 173). Of these, 45.7% discussed issues relating to clinics, whereas issues relating to community health workers and nurses were covered by 42.8% and 34.1% of news stories, respectively. The number of news stories discussing infectious diseases (55.5%) was more than twice the number discussing non-communicable diseases (21.4%). HIV/AIDS/TB illness- and service-related issues were covered by 54.3% of news stories and social determinants of health by 22%. Issues relating to how healthcare is organised to deliver services to the people received substantial coverage in the print media, with 72.8% discussing delivery arrangements, 72.3% governance arrangements, and 55% financial arrangements. A small fraction of news stories (7.5%) discussed research studies but none discussed a systematic review.

**Conclusion:**

Our study underscores the potential role of media analyses in illuminating patterns in print media coverage of health issues. It also shows that an understanding of coverage of health research evidence could help spur efforts to support the climate for evidence-informed health policymaking. Researchers in low- and middle-income countries need to be more proactive in making use of media analyses to help illuminate health related issues that require the attention of health policymakers, stakeholders and reporters, and to identify potential areas of research.

**Electronic supplementary material:**

The online version of this article (doi:10.1186/s12961-015-0051-6) contains supplementary material, which is available to authorized users.

## Background

The concept of primary healthcare (PHC) developed by WHO in the Alma Ata Declaration is a broad philosophy and key strategy for achieving equitable health outcomes [1,2]. The PHC approach encompasses comprehensive care, active community participation and empowerment, equity, intersectoral collaboration, and use of appropriate technology [1]. A key feature of the PHC approach was the promotion and expansion of community health worker (CHW) or lay health worker programmes in low- and middle-income countries (LMICs) in the 1970s and 1980s [2,3]. However, there was a decline in enthusiasm for PHC initiatives in the late 1980s to early 1990s because of challenges with funding, lack of adequate training and supervision, lack of appropriate incentives, and high turnover among CHWs, among other factors [4,5]. In the last decade, there has been a resurgence of interest in PHC in response to the rapid expansion in both government and international donor funding for vertical healthcare initiatives such as HIV/AIDS and child survival programmes [5,6]. The WHO has also promoted home and community-based care and the concept of task-shifting to deal with health worker shortages in LMICs [7,8]. These shifts in emphasis have also served to increase the involvement of CHWs in healthcare delivery at the primary care level [7,9].

PHC has been shown to improve access to healthcare services and population health outcomes [10,11]. A number of LMICs that have adopted the PHC approach on a national scale, such as Thailand and Brazil, have achieved remarkable improvements in population health [10,11]; Thailand was able to achieve universal coverage of immunisation and skilled birth attendance [9]. A systematic review of interventions by CHWs mainly in western countries showed that, when compared to usual care, CHW programmes were more effective in promoting immunisation uptake and improving outcomes for acute respiratory infections and malaria [12]. In addition, CHW programmes were found to be effective in increasing breastfeeding and decreasing mortality rates in the elderly through the provision of health services [12]. In sub-Saharan Africa, a recent systematic review of CHWs in HIV care showed that CHWs improved the reach, uptake and quality of HIV services as well as the quality of life and retention in care for people living with HIV. CHWs also helped reduce waiting times in clinics, streamline patient flow and reduce the workload of health workers [13].

In South Africa, a number of policy developments have shaped PHC since the beginning of democratic rule (Table 1). In 1994, the newly elected government unveiled a national health plan and a district health system premised on the principles of PHC. However, many PHC initiatives in the country suffered from inadequate leadership and financial support from the government and from donor fatigue [5,14,15].

**Table 1:** **Timeline of key policy developments about primary healthcare (PHC) relevant to South Africa, 1994–2012**

**Date**	**Key policy developments**
1994	First democratic elections; African National Congress (ANC) government assumes power
1994	ANC unveils National Health Plan premised on a primary health care philosophy
1994	Government introduces a school nutrition programme in schools located in poor communities
1994	President Nelson Mandela announces the introduction of free health care in public health facilities for pregnant women and children under the age of 6 years
1995	Department of Health releases the implementation strategy for a decentralised district-based health system
1995	Government embarks on the building of primary health care facilities across the country
1996	Government extends free primary health care to all users of public health facilities
2003	Department of Health develops Community Health Workers Policy Framework
2004	Department of Health introduces a publicly funded antiretroviral programme
2004	Department of Health introduces umbrella term ‘Community Health Worker’ for all community workers in the health sector and adopts the National Community Health Worker Policy Framework
2004	National Health Act legislates the establishment of the district health system as a vehicle for the delivery of primary health care throughout the country
2004	The Department of Public Works launches the Expanded Public Works Programme Social Sector Plan which begins s skills training and learnerships for community caregivers
2006	The World Health Organization proposes the training of community health workers in its AIDS and health workforce plan
2008	The World Health Organization, in collaboration with UNAIDS and PEPFAR, publishes global recommendations and guidelines for theimplementation of task shifting among health workforce teams
2009	Department of health develops the Community Care Worker Management Policy Framework
2009	Department of public works launches second phase of the Expanded Public Works Programme which continues to provide employment,skills training and learnerships for community caregivers
2010	Health Minister releases discussion document on Primary Health Care Re-engineering
2011	Health Minister releases green paper on the National Health Insurance initiative, which includes primary health care reengineering
2012	Government rolls out ward-based primary health care teams across pilot sites as part of the Primary Health Care Re-engineering initiative
2012	Department of Basic Education and Social Development launches the new national integrated School Health Policy, an element of the Primary Health Care Re-engineering initiative

In the late 1990s, the South African government began to support initiatives aimed at reducing the burden of HIV/AIDS on the public health system by funding non-governmental organizations providing home- and community-based care for people living with HIV/AIDS [6,16]. Yet, it was not until 2001 that a draft policy guideline on home/community-based care was published by the Department of Health [15,17,18].

Since 2004, there has been increased government support and funding of various PHC initiatives, which has led to considerable growth in the number of CHWs in the country. These comprise generalist CHWs as well as those working in home- and community-based care and in clinics as specialist HIV/TB workers [5,15,19]. In an attempt to improve PHC services in the country, the South African government, in 2010, prepared a formal discussion document on an initiative to re-engineer PHC as a key component of the national health insurance (NHI) initiative and released a green paper on the topic in 2011 [20,21]. A key feature of the policy is the formalization of CHWs. The NHI initiative has spurred a lot of discussion in the media about healthcare financing and health systems reform at the PHC level and this has brought into sharp focus the need to examine issues relating to the role of the media in discussions about PHC in South Africa (see Table 1 for key policy developments).

One of the major potential influences on policymaking and decision making, more generally, is the media coverage of issues and events [22–24]. The news media is located at the interface between public and policy agendas, and plays a major role in setting policy agendas [25] as well as providing a window into issues concerning the general population [26,27] and helping influence policy agendas by focusing public attention on particular issues at the expense of others [26–29]. Therefore, the media could play a role in framing policy debates about major health reform issues [23,26,30]. In addition, by covering policy issues in the health sector, the media could help inform research agendas by providing researchers with information on themes and issues that need research attention [31].

The news media can also help shape the climate for evidence-informed health policymaking, which refers to the systematic and transparent use of research evidence in government decisions about health [32,33]. By bringing health research evidence to the attention of policymakers and stakeholders, the news media can serve to inform policymakers about research results that could help inform policymaking. For example, media reporting of evidence from evaluations of healthcare interventions, particularly systematic reviews of such evaluations, could provide policymakers with the most robust form of evidence for informing policy decisions about the allocation of resources and decisions on how best to deliver, pay for and govern these services [32–34]. The coverage of issues by the news media is determined to a large extent by ‘the gatekeepers’, who apply a set of criteria to judge the ‘newsworthiness’ of issues. Gatekeeping is the process through which the vast array of potential news is narrowed down into the small volume that is prioritized by the news media [35]. Potential news items are moved along, halted or discarded as they pass through news channels from the source to a reporter to a number of editors [35-37]. Through the gatekeeping process, media organizations help determine what is and what is not covered, thereby influencing policy agendas [36,37].

Given the potential role of the media in shaping decision making, understanding media coverage of issues relating to PHC over a period of time could be critical in informing PHC policies in South Africa. However, there is little research about print media coverage of PHC in South Africa; we did not find any published study on this theme. In the present study, we explore how the print media covers PHC and related research evidence.

## Methods

We used LexisNexis Academic – the world’s largest online collection of news services – to search for news stories for the print media analysis. LexisNexis contains functions for identifying newspapers from different countries and to perform searches for news stories with various terms/keywords. Previous studies have used LexisNexis to conduct media analyses on health issues [30,38]. Our search was restricted to newspapers covered in the *Major World Publications* component of LexisNexis Academic News. The searches were conducted in July and August 2013.

### Newspaper search and selection strategy

We developed a search strategy that enabled us to retrieve newspapers available in LexisNexis that were relevant to the purpose of the study using a set of inclusion criteria. Specifically, the inclusion criteria were newspapers that were (1) classified as a South African newspaper in LexisNexis; (2) published in English; (3) published for a fairly broad readership (for example, a newspaper with exclusive focus on defence issues was excluded because we felt it is not widely read by the general public); and (4) covered for at least one full year (January–December) in LexisNexis. The latter was also necessary for calculating trends in coverage over the study period. A total of 25 newspapers met the inclusion criteria and were therefore included in the subsequent analyses (Table 2).

**Table 2:** **Newspaper characteristics and period covered in analysis**

**Geographical circulation of newspapers**	**Newspapers**	**Frequency**	**Publisher**	**LexisNexis coverage (Start–end dates)**	**Period covered in analysis**	**Number of years covered in analysis**	**Newspaper circulation (2012)**
National newspapers							
	Mail & Guardian	Weekly	M & G Media Ltd., Johannesburg	Jan 2010–current	2010/01–2012/12	3	48,999
	Post^a^	Weekly	Independent Newspapers, Johannesburg	Jan 2007– current	2007/01–2012/12	3	44,683
	Star (The)	Mon–Sat	Independent Newspapers, Johannesburg	July 2006–current	2007/01–2012/12	6	102,244
	Sunday Independent (The)	Weekly	Independent Newspapers, Johannesburg	Jan 2007–current	2007/01–2012/12	6	35,263
	Sunday Times	Weekly	Avusa Media Ltd., Johannesburg	Jan 1997–current	1997/01–2012/12	16	449,799
	Sunday Tribune	Weekly	Independent Newspapers, Johannesburg	Jan 2007–current	2007/01–2012/12	6	71,675
	Sunday World	Weekly	Avusa Media Ltd., Johannesburg	Oct 2008–current	2009/01–2012/12	4	130,656
	Times (The)	Weekly	Avusa Media Ltd., Johannesburg	Sept 2008–current	2009/01–2012/12	4	146,956
Provincial newspapers							
Eastern Cape	Algoa Sun	Monthly	Avusa Media Ltd., Johannesburg	Aug 2010–current	2011/01–2012/12	2	20,337
	Daily Dispatch	Mon–Sat	Avusa Media Ltd., Johannesburg	Sept 2008–current	2009/01–2012/12	4	26,339
	Go! & Express	Weekly	Avusa Media Ltd., Johannesburg	Aug 2010–current	2011/01–2012/12	2	37,454
	Herald (The)	Mon–Fri	Avusa Media Ltd., Johannesburg	Sept 2008–current	2009/01–2012/12	4	22,079
	Our Time	Weekly	Avusa Media Ltd., Johannesburg	Sept 2010–current	2011/01–2012/12	2	2,323
	Representative (The)	Weekly	Avusa Media Ltd., Johannesburg	Aug 2010–current	2011/01–2012/12	2	5,642
	Talk of the Town	Weekly	Avusa Media Ltd., Johannesburg	Aug 2010–current	2011/01–2012/12	2	2,784
	Weekend Post	Weekly	Avusa Media Ltd., Johannesburg	May 2009–current	2010/01–2012/12	3	21,694
Gauteng	Pretoria News	Mon–Sat	Independent Newspapers, Johannesburg	July 2006–current	2007/01–2012/12	6	17,576
	Sowetan	Daily	Avusa Media Ltd., Johannesburg	Sept 2008–current	2009/01–2012/12	4	100,349
KwaZulu-Natal	Business Day	Daily	Avusa Media Ltd., Johannesburg	Aug 1997–current (some from Jan 1997)	1998/01–2012/12	15	35,149
	Daily News	Daily	Independent Newspapers, Johannesburg	July 2006–current	2007/01–2012/12	6	30,743
	Independent Saturday (The)	Weekly	Independent Newspapers, Johannesburg	April 2007–current	2008/01–2012/12	5	43,011
	Mercury	Daily	Independent Newspapers, Johannesburg	July 2006–current	2007/01–2012/12	6	29,761
Western Cape	Cape Argus/Argus weekend	Daily	Independent Newspapers, Johannesburg	July 2006–current	2007/01–2012/12	6	32,337/60,383
	Cape times	Daily	Independent Newspapers, Johannesburg	July 2006–current	2007/01–2012/12	6	34,627
	West Cape News	Daily	Africa News Service Inc., USA	Jan 2010–current	2010/01–2012/12	3	^b^

^a^Has circulation only in Gauteng and KwaZulu-Natal provinces.

^b^Circulation not available.

We had intended to begin our search of newspapers eligible for inclusion from 1994 when the African National Congress government began democratic rule, which represents the beginning of a distinct era in health policy and a period of marked change in policy direction for PHC in South Africa [20]. However, preliminary searches in LexisNexis revealed that there was no South African newspaper coverage from 1994–1996. Our searches therefore covered all news stories that were available in LexisNexis over the 16-year period 1997–2012, inclusive (Table 2).

### Search strategy for news stories

Next, we proceeded to search for news stories from the 25 newspapers using specific terms related to PHC in South Africa. In order to retrieve as many news stories on PHC as possible, we decided to include all news stories containing specific terms/concepts related to PHC that are generally used in the literature on PHC in South Africa rather than restricting our search to only the term ‘primary healthcare’. Additionally, rather than searching for news stories that contained any of these PHC related terms in their titles, we chose to search for news stories that had used these terms in both their titles and full texts. Lastly, we did not restrict our search to any select sections of the newspapers but instead searched all sections of all newspapers that met our inclusion criteria.

We used two broad categories of concepts for our searches. First, we used broad concepts widely known as being related to PHC in South Africa. These include PHC, community-based care, community-based healthcare, and home-based care. Second, we used terms that represented the broad scopes of practice/cadres of the diverse PHC workers in South Africa commonly used in the literature and in government policy documents [6,14,15,17,18,39]. The terms are CHW, lay health worker, home-based caregiver/carer, community care worker, community caregiver, and volunteer caregiver/carer. For each of these terms, we used variations in the terms in order to retrieve all possible related news stories. For example, ‘primary health care’ versus ‘primary healthcare’ and ‘home-based care’ versus ‘home based care’. We also used both singular and plural forms of each term representing the cadres of PHC workers such as ‘community caregiver’ and ‘community caregivers’.

### Selection and analysis of news stories

**Figure 1:**
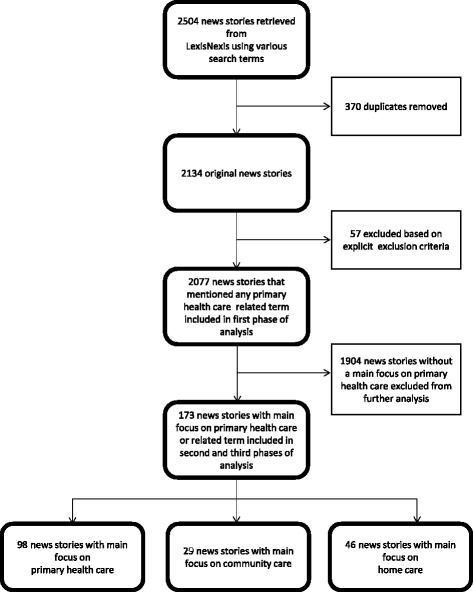
**Flow chart showing sample selection process.**

We retrieved a total of 2,504 news stories from the 25 South African newspapers (Additional file 1). In order to remove news stories that were deemed not relevant to the purpose of our study, we developed a set of explicit exclusion criteria. First, we excluded all duplicate news stories. Second, we excluded all stories that did not have a focus on South Africa, were about PHC of animals or were written in a language other than English. Only 2,077 news stories remained after we applied our explicit exclusion criteria (Figure 1).

We developed a three-phase strategy to analyse all 2,077 news stories that met our inclusion criteria. First, we developed criteria for coding and classifying news stories into three categories based on the foci of the news stories (Additional file 2). The criteria were set to help us decide whether each of the news stories had a (1) main focus on PHC (for further analysis in the second phase and full coding in the third phase, and inclusion in news story counts), or (2) secondary focus on PHC (for inclusion in news story counts only, or (3) whether PHC was only briefly mentioned in the news story (for inclusion in news story counts only). We then proceeded to read all news stories in full and to use our criteria to code each of the news stories into these three categories. In total, 173 news stories were classified as having a main focus on PHC and subsequent analyses (the second and third phases) were conducted only on these 173 news stories (Additional file 3).

In the second phase, we analysed news stories classified as having a main focus on PHC for basic characteristics like newspaper name, geographical area of circulation, type of news story, year of publication, length of news story, and news focus. In the third phase, we developed a data extraction form to help in coding all news stories. The form was developed in an iterative manner and discussed extensively and revised by the authors before it was applied. In order to identify concepts/terms for developing the extraction form, we first listed terms/concepts used in PHC in South Africa. Second, we extracted more terms from a rapid review of the literature on PHC in South Africa. Third, we identified even more terms/concepts by reading a random sample of 50 news stories. We then used these terms/concepts to develop codes for issues and themes used in the form. The form contained codes for identifying and classifying concepts related to the following themes: where PHC is provided or its consequences are felt, by whom PHC is provided, with what focus PHC is discussed, health system arrangements and implementation strategies, and use of research evidence.

In order to identify codes for issues relating to health systems arrangements and implementation strategies, we reviewed and applied the taxonomy developed for Health System Evidence [40,41]. Lastly, we developed codes for identifying different kinds of research evidence reported in news stories.

### Inter-rater agreement

In the first phase of analysis, two independent raters performed the coding of the news stories. One rater coded all 2,077 news stories that met our inclusion criteria while a second rater coded a random sample (350 or about 16%) of the news stories. We achieved a Kappa score of 0.79. One of the authors helped in making a decision in cases where consensus could not be reached. In the second phase, one of the authors extracted the basic characteristics of the news stories and a second coder checked for accuracy. In the third phase, the two independent raters coded all (n = 173) news stories, using an iterative method. They met regularly to work through and resolve discrepancies arising from the coding. The second rater was trained by the first and both worked on a sample of trial news stories before proceeding to code the remaining stories.

## Results

**Figure 2:**
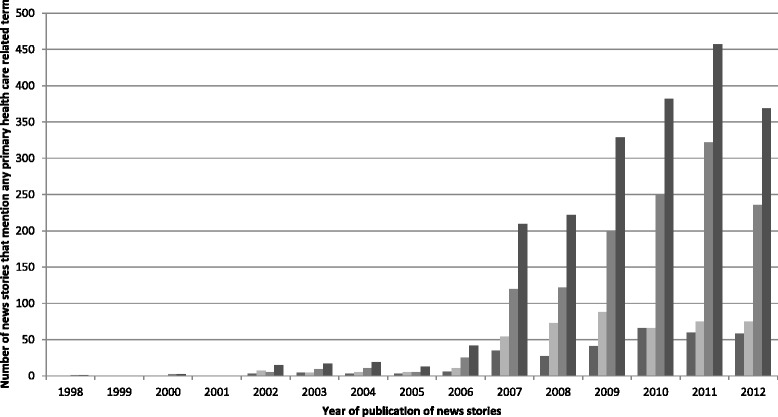
**Number of news stories that mention any primary healthcare-related term.**

Of the 2,077 news stories that met our inclusion criteria, 1,308 mentioned the term PHC, 463 mentioned a term related to home care, and 307 mentioned a term related to community care (Figure 2). News stories that had only a brief mention of PHC accounted for by far the greatest number (n = 1,796, 86.5%), while 108 (5.2%) news stories had a secondary focus on PHC. A total of 173 (8.3%) news stories had their main focus on PHC (Additional file 3).

**Figure 3:**
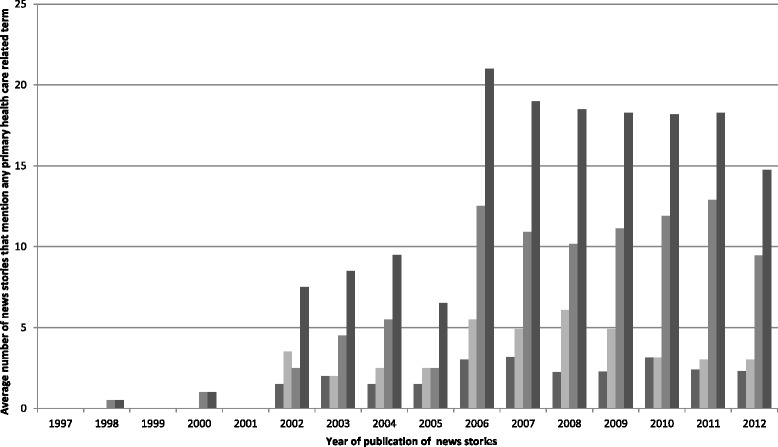
**Average number of news stories that mention any primary healthcare-related term per newspaper.**

Figure 3 shows that the highest average number of news stories mentioning any PHC term over a 16-year period was in 2006.

**Figure 4:**
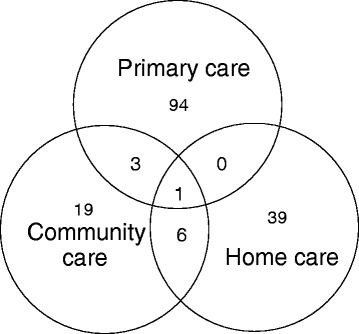
**Foci of news stories that mention primary healthcare-related term.**

Of the 173 news stories, 98 (56.6%) had their main focus on the term PHC, 46 (26.6%) had their main focus on home care, and 29 (16.8%) had their main focus on community care. The highest number of overlaps (n = 6, 3.5%) were between news stories focusing on community care and home care (Figure 4).

Table 3 shows the characteristics of the 173 news stories with a main focus on PHC. The majority (92%) of the news stories with a PHC focus were published by only two media organizations. Most (140, 80.9%) of the news stories were published in provincial newspapers, while news stories published in national newspapers accounted for the remaining 33 (19.1%). Of the 173 news stories published in national and provincial newspapers, the largest grouping (43, 24.9%) were published in newspapers from the Eastern Cape, followed by the Western Cape (41, 23.7%) and Gauteng (35, 20.2%). Of the total number of news stories published in both national and provincial newspapers, Cape Argus/Argus Weekend and the Herald had the highest number (32, 18.5% and 31, 17.19%) of news stories followed by the Sowetan (22, 12.7%).

**Table 3:** **Characteristics of news stories with main focus on primary healthcare (n = 173)**

**Variable**	**Sub-variable**	**Total**	**Percent**
**Newspaper source**			
**National newspapers**		**33**	**19.1**
	Star (The)	15	8.7
	Sunday Times	6	3.5
	Sunday Tribune	5	2.9
	Times (The)	3	1.7
	Post	3	1.7
	Sunday Independent (The)	1	0.6
	Sunday World	0	0.0
	Mail & Guardian	0	0.0
**Provincial newspapers**		**140**	**80.9**
	**Eastern Cape newspaper articles**	**43**	**24.9**
	Herald (The)	31	17.9
	Daily Dispatch	9	5.2
	Weekend Post	2	1.2
	Representative (The)	1	0.6
	Algoa Sun	0	0.0
	Go! & Express	0	0.0
	Our Times	0	0.0
	Talk of the Town	0	0.0
	**Gauteng newspaper articles**	**35**	**20.2**
	Sowetan	22	12.7
	Pretoria News	13	7.5
	**KwaZulu-Natal newspaper articles**	**21**	**12.1**
	Daily News	9	4.6
	Business Day	9	5.2
	Mercury (The)	3	1.7
	**Western Cape newspaper articles**	**41**	**23.7**
	Cape Argus/Argus weekend	32	18.5
	Cape Times	7	4.0
	West Cape News	2	1.2
**Publisher**	Independent Newspapers Ltd.	88	50.9
	Avusa Media Ltd.	83	48.0
	African News Service Inc.	2	1.2
	M & G Media Ltd.	0	0
**News focus**	Primary healthcare	98	56.6
	Community care	29	16.8
	Home care	46	26.6
**Type of news item**	General news	72	41.6
	Health	47	27.2
	Not specified	10	5.8
	Labour	8	4.6
	Economy, business & finance	6	3.5
	Human interest	6	3.5
	Social issues	6	3.5
	Opinion & editorial	6	3.5
	Entertainment	4	2.3
	Politics	4	2.3
	Education	2	1.2
	Life	1	0.6
	Dispatches	1	0.6
**News length, words count**	1–250	43	24.9
	251–500	74	42.8
	501–1000	48	27.7
	1001–2000	8	4.6
**Year of publication**	1998–2003	0	0
	2006	3	1.7
	2007	14	8.1
	2008	15	8.7
	2009	18	10.4
	2010	34	19.7
	2011	44	25.4
	2012	45	26.0

All the community newspapers in our sample (Algoa Sun, Go! & Express, Talk of the Town and The Representative) are published in the Eastern Cape and only one of them (The Representative) had one news story on PHC. It is also striking that two national newspapers (Mail & Guardian and Sunday World), with a combined circulation of about 180,000, did not have any news story focused on PHC. With respect to the type of news item, the majority were published as general news stories (72, 41.6%) and health news (47, 27.2%). Most (74, 42.8%) of the news stories had a length of 251–500 words. More than half of the news stories were published in two years (2011 and 2012).

Of the 173 news stories discussing issues relating to where PHC is provided (Table 4), the majority (79, 45.7%) discussed issues relating to clinics while 39 (22.5%) discussed issues relating to hospitals. Most of these news stories discussed the implications of PHC for hospitals and 12 (6.9%) news stories discussed issues relating to schools.

**Table 4:** **Number of news stories with a main focus on primary healthcare (PHC), by specific issues discussed**

	**Focus**	**Primary healthcare**	**Community care**	**Home-based care**	**Total**	**Percent**
Where PHC is provided or its consequences are felt	**All clinics**	**61**	**6**	**12**	**79**	**45.7**
	PHC clinics/facilities	35	3	4	42	24.3
	Mobile clinics	11	2	3	16	9.2
	Health trains	5	1	1	7	4.0
	Community-based health care centres	4	2	3	9	5.2
	Mobile dental clinics	4	0	0	4	2.3
	Fixed clinics	3	0	1	4	2.3
	**All hospitals**	**28**	**5**	**6**	**39**	**22.5**
	Hospitals	19	4	6	29	16.8
	Day hospitals	2	2	1	5	2.8
	**All other settings**	**9**	**4**	**8**	**21**	**12.1**
	Schools	9	2	1	12	6.9
	Hospices	0	2	10	12	6.9
	Workplaces	2	0	0	2	1.2
By whom PHC is provided	**All community health workers**	**14**	**23**	**37**	**74**	**42.8**
	Home-based carer/caregivers	2	5	28	35	20.2
	Community care workers	7	12	4	23	13.3
	Volunteer caregivers	3	5	14	22	12.7
	Community caregivers	4	5	6	15	8.7
	Directly observed treatment supporters	1	4	3	8	4.6
	Lay counsellors	1	2	1	4	2.3
	**All nurses**	**40**	**8**	**11**	**59**	**34.1**
	PHC nurses	18	3	0	21	12.1
	Professional nurses	12	0	3	15	8.7
	Psychiatric nurses	3	1	0	4	2.3
	Senior professional nurses	2	0	1	3	1.7
	Enrolled nursing assistants	1	0	2	3	1.7
	Midwives	2	0	0	2	1.2
	Enrolled nurses	2	0	0	2	1.2
	Nurse practitioners	1	0	0	1	0.6
	Paediatric nurses	1	0	0	1	0.6
	**All doctors**	**23**	**8**	**3**	**34**	**19.7**
	Specialists	4	2	1	7	4.0
	Family physicians	3	0	0	3	1.7
	Dentists	2	1	0	3	1.7
	Obstetricians & gynaecologists	2	0	0	2	1.2
	Paediatricians	2	0	0	2	1.2
	**Pharmacists & allied workers**	**6**	**2**	**0**	**8**	**4.6**
	Pharmacists	6	2	0	8	4.6
	Pharmacy assistants	2	0	0	2	1.2
	**All other care providers**	**8**	**2**	**4**	**14**	**8.1**
	Traditional healers	3	2	3	8	4.6
	Social workers	1	1	1	3	1.7
	Psychologists	2	0	0	2	1.2
With what focus is PHC discussed?	**Infectious diseases & services**	**40**	**17**	**39**	**96**	**55.5**
	HIV/AIDS/TB & services	39	17	38	94	54.3
	Treatment & care	22	10	19	51	29.5
	Counselling & testing	15	2	9	26	15.0
	Prevention	14	0	10	24	13.9
	Screening	10	0	5	15	8.7
	Sexually transmitted infections	6	0	8	14	8.1
	**Chronic conditions & services**	**28**	**17**	**7**	**37**	**21.4**
	Mental healthcare/services	10	2	4	16	9.2
	Cancer & cancer treatment services	8	0	3	11	6.4
	Hypertension	11	0	0	11	6.4
	Cardiac disease	6	0	0	6	3.5
	Diabetes	4	0	2	6	3.5
	**Reproductive health**	**19**	**0**	**2**	**19**	**11.0**
	Maternity services	15	0	2	17	9.8
	Family planning	10	0	2	12	6.9
	**Other forms of care**	**39**	**4**	**20**	**63**	**36.4**
	Child health	33	1	8	42	24.3
	Orphan & vulnerable children care	21	1	0	22	12.7
	Maternal & child health	1	1	12	14	8.1
	Oral health	13	1	0	14	8.1
	Palliative care	1	2	8	11	6.4
	Eye health	8	0	0	8	4.6
	Elderly care	2	1	3	6	3.5
	Ear nose & throat	3	0	0	3	1.7
	Rape/sexual assault services	3	0	0	3	1.7
	**Social determinants of health**	**22**	**1**	**15**	**38**	**22.0**
	Nutrition	14	1	12	27	15.6
	Poverty	9	1	13	23	13.3
	Unemployment	8	0	7	15	8.7
	Food security	6	0	3	9	5.2
	Education	2	0	2	4	2.3
	Water	3	0	1	4	2.3
	Housing	1	0	0	1	0.6

With regards to health care professionals working in PHC, the majority of news stories discussed issues relating to CHWs (74, 42.8%) followed by nurses (59, 34.1%) and doctors (34, 19.7%). In terms of the health services focus, the number of news stories discussing at least one infectious disease was more than twice the number discussing at least one non-infectious disease (96, 55.5% and 37, 21.4%). HIV/AIDS/TB were the most discussed infectious diseases (94, 54.3%) while mental health (16, 9.2%) was the most discussed non-infectious disease. Issues relating to child health were discussed by 42 (24.3%) news stories while issues relating to orphan and vulnerable child care were discussed by 22 (12.7%) news stories. Only 3 (1.7%) news stories discussed rape or sexual violence. Issues relating to social determinants of health were discussed by 38 (22.0%) news stories, and issues relating to nutrition (27, 15.6%) and poverty (23, 13.3%) were the most discussed social determinants of health.

As shown in Table 5, issues relating to delivery arrangements (126, 72.8%) and governance arrangements (125, 72.3%) were each discussed by roughly three-fourths of the news stories. Issues relating to financial arrangements were discussed by 95 (54.9%) news stories. Accountability of the state sector’s role in financing and delivery of health services was the single most covered news item discussed by 109 (63.0%) news stories. Issues relating to the NHI (taxation) were also discussed by 17 (9.8%) stories. However, only a few (15, 8.7%) discussed implementation strategies.

**Table 5:** **Number of news stories with a main focus on primary healthcare by broad health systems arrangement focus and reference to research evidence**

	**Focus**	**Primary healthcare**	**Community care**	**Home-based care**	**Total**	**Percentage**
**Health systems arrangements**	**Governance arrangements**	**77**	**22**	**26**	**125**	**72.3**
	***Policy authority***					
	Centralization/decentralization of policy authority	33	3	0	36	20.8
	Accountability of the state sector’s role in financing and delivery	68	20	21	109	63.0
	***Organizational authority***					
	Management approaches	17	0	1	18	10.4
	Partnership/networks	13	2	4	19	11.0
	***Professional authority***					
	Training & licensure requirements	18	9	7	34	19.7
	Scope of practice	8	4	3	15	8.7
	Strike/job action	6	7	8	21	12.1
	**Financial arrangements**	**57**	**15**	**23**	**95**	**55.0**
	***Financing systems***					
	Taxation	14	2	1	17	9.8
	User fees	5	0	1	6	3.4
	Donor contribution	6	2	16	24	13.9
	***Funding organizations***					
	Funding organizations	41	14	8	63	36.4
	Targeted payments/penalties	2	1	0	3	1.7
	***Remunerating providers***					
	Capitation	1	0	0	1	0.6
	Salary	10	7	12	29	16.8
	Voucher	1	0	0	1	0.6
	**Delivery arrangements**	**83**	**21**	**22**	**126**	**72.8**
	***How care is designed to meet consumers’ needs***					
	Availability of care	36	7	9	52	30.0
	Timely access to care	25	3	4	32	18.5
	Culturally appropriate care	2	0	1	3	1.7
	Package of care	15	1	1	17	9.8
	***By whom care is provided***					
	Need, demands & supply	21	2	1	24	13.9
	Recruitment, retention & transitions	14	1	1	16	9.2
	Performance management	1	0	0	1	1.7
	Provider satisfaction	18	11	11	40	23.1
	Health & safety	2	2	4	8	4.6
	Role performance	1	0	0	1	0.6
	Role expansion/extension	15	3	3	21	12.1
	Task shifting/substitution	2	0	0	2	1.2
	Multidisciplinary teams	4	3	2	9	5.2
	Volunteers	1	1	1	3	1.7
	Training	16	6	7	29	16.8
	Support	3	2	2	7	4.0
	Workload/workflow/intensity	13	3	1	17	9.8
	***Where care is provided***					
	Site of service delivery	25	3	4	32	18.5
	Physical structure, facilities & equipment	23	3	3	29	16.8
	Organizational scale	5	0	1	6	3.5
	Integration of services	8	2	4	14	8.1
	Continuity of care	7	0	0	7	4.0
	Outreach	16	2	1	19	11.0
	**With what supports is care provided?**					
	Health records	1	0	0	1	0.6
	Electronic health record	1	0	0	1	0.6
	Other information and communication technologies that support individuals who provide care	1	0	0	1	0.6
	**Implementation strategy**	**9**	**3**	**3**	**5**	**8.6**
	***Consumer-targeted strategy***					
	Information/education provision	9	2	3	14	8.0
	Behaviour change	9	2	3	14	8.0
	Skills development	1	2	1	4	2.3
	Personal support	1	0	0	1	0.6
**Research evidence**	**Research studies**	**9**	**2**	**2**	**3**	**7.5**
	Studies	8	2	2	12	6.9
	Refereed journal articles	1	0	0	1	0.6
	Systematic review study	0	0	0	0	0.0

A small number of news stories (13, 7.5%) discussed issues relating to research studies. Five of these news stories discussed the same study which was published in the South African Health Review, a non-refereed publication. Three of the remaining news stories discussed student dissertations and three did not specify whether or not the study discussed was published. Only one of the news stories discussed a study published in a scientific journal and none of the stories discussed a systematic review.

Table 6 shows the results of χ^2^ tests to investigate associations between newspaper characteristics and health system arrangement variables. Newspaper coverage (national vs. provincial), provinces/area covered by newspaper, newspaper section where the news story was published, news story length, and news story focus were associated with at least one of the health system variables. News story focus was associated with 12 health system variables. These include three governance arrangement variables, two financial arrangement variables and seven delivery arrangement variables.

**Table 6:** **Association between newspaper characteristics and coverage of health-system arrangements (using a χ** ^**2**^ **test of independence)** ^a^

**Variable**	**Governance arrangements**				**Financial arrangements**		**Delivery arrangements**					
	**De/centralization of policy authority**	**Accountability of the state sector’s role in financing and delivery**	**Training & licensure requirements**	**Strike/job action**	**Donor contributions**	**Funding organizations**	**Availability of care**	**Timely access to care**	**Package of care**	**Needs, demands & supplies**	**Recruitment, retention & transitions**	**Provider satisfaction**
**Coverage**												
National	6.1^b^	51.5	18.23	18.2	----	----	36.4	21.2	15.2	15.2	6.1	18.2
Provincial	24.3	65.7	20.0	10.7	----	----	28.0	17.9	8.0	13.6	9.3	23.6
**Province covered**												
National	6.1^d^	51.5	18.2	----	----	----	----	----	----	----	----	----
Eastern Cape	41.9	69.8	11.6	4.7	----	----	----	----	----	----	----	----
Gauteng	8.6	51.4	11.4	4.3	----	----	----	----	----	----	----	----
KwaZulu-Natal	23.8	66.7	23.8	4.8	----	----	----	----	----	----	----	----
Western Cape	19.5	73.2	34.1	17.1	----	----	----	----	----	----	----	----
**News type**					----	----						
General news	----	63.9^d^	----	----	----	----	----	----	----	----	----	----
Health news	----	76.6	----	----	----	----	----	----	----	----	----	----
Not specified	----	70.0	----	----	----	----	----	----	----	----	----	----
Labour & economy	----	64.3	----	----	----	----	----	----	----	----	----	----
Social issues & human interest	----	22.2	----	----	----	----	----	----	----	----	----	----
Opinion/editorial, politics & education	----	58.3	----	----	----	----	----	----	----	----	----	----
**News length**												
1–250	16.3	51.2	14.0	11.6	----	----	39.5^b^	----	----	----	----	----
251–500	21.6	67.6	20.3	10.8	----	----	28.4	----	----	----	----	----
501–1000	22.9	64.6	20.8	16.7	----	----	39.6	----	----	----	----	----
1001–2000	25.0	75.0	37.5	0.0	----	----	75.0	----	----	----	----	----
**News focus**												
Primary healthcare	32.7^d^	68.4^c^	16.3^d^	6.1^c^	7.1^d^	39.8^b^	33.7^c^	24.5^b^	15.3^b^	20.4^c^	13.3^b^	16.3^c^
Community-based care	13.8	72.4	41.4	24.1	3.4	48.3	31.0	17.2	3.4	13.8	6.9	41.4
Home-based care	0.0	45.7	13.0	17.4	32.6	21.7	10.9	6.5	2.2	0.0	0.0	23.9

^a^χ^2^ results are shown for each variable for which a sufficiently large sample was available to test the association (i.e. that will yield a cell size ≥5) and only for those health-system arrangement variables where at least one association is statistically significant.

^b^ *p* < 0.05.

^c^ *p* < 0.01.

^d^ *p*< 0.001.

Issues relating to centralization and decentralization of policy authority were more likely to be covered by Eastern Cape newspapers than by newspapers published in other provinces or national newspapers. Two newspaper characteristics (newspaper publisher and year of publication of news story; not shown on the table) were not associated with any of the health system variables.

## Discussion

### Principal findings

We note a steady increase in the frequency of news stories that mentioned PHC from 2006 to 2012 with a peak in 2011. Although only two newspapers were included in our sample from 1998 to 2005, it is striking that there was no newspaper coverage of PHC in 2001 when the policy guideline for home and community-based care was developed. The average number of news stories per newspaper shows an increase in coverage of PHC from 2002 to 2004, which corresponds with key policy developments about PHC during this period. While some of these stories covered issues about home and community-based care, none of them made reference to any of the key policy developments during this period. It is not clear, however, what was responsible for the spike in average number of news stories per newspaper in 2006 and 2007 as none of the news stories discussed the WHO health workforce plan, which was the key policy development in 2006.

Provincial newspapers covered a lot more stories on PHC than national newspapers. This appears to reflect the smaller number of national newspapers in our sample. It could also be because, in South Africa, the implementation of PHC occurs at the provincial and district levels. Issues relating to the implementation of health policies are therefore more likely to be covered by provincial newspapers than national newspapers. This argument is supported by the finding that provincial newspapers (and particularly Eastern Cape newspapers) were significantly more likely to cover stories on centralization and decentralization of policy authority. Most of these stories were covered by The Herald and were about tensions between the Eastern Cape provincial department of health and the municipalities regarding a policy requiring municipalities to hand over the running of PHC facilities and services to the provincial government.

The highest frequency of news stories with a main focus on PHC were in 2011 and 2012 and this corresponds with the peaks observed in the total number of news stories that mentioned PHC. The release of a discussion document on the initiative to re-engineer PHC in 2010 and a green paper on the NHI in 2011 seem to have generated increased public debates, which gained prominence in the print media during 2011 and 2012. An analysis of the news stories during this period showed that they comprised coverage of conference key note presentations by policymakers and public comments by researchers, journalists and the general public on the PHC re-engineering and the NHI initiatives. In addition, there were many news stories discussing issues relating to the value of CHWs to the health system as well as their management and remuneration.

It is also important to note that a large number of the news stories were published as general news items compared with those published as health news. HIV/AIDS/TB and mental health issues were the most discussed health issues, while issues relating to rape or sexual violence were rarely discussed. With regards to health systems arrangement issues, governance arrangement and delivery arrangement issues were the two most discussed issues. Accountability of the state sector’s role in financing and delivery was the most discussed governance arrangement issue and was significantly more likely to be published as health news than as any other news item.

Despite several major policy developments and several prominent publications on PHC in the 16-year study period, there was limited coverage of health research evidence about PHC and most of the stories on health research evidence were published in non-referred journals. There was also very little discussion about the studies covered in the new stories. None of the news stories covered any systematic review.

### Strengths and limitations

Our study has a number of strengths. Firstly, we used LexisNexis Academic, the largest and most comprehensive database of newspaper articles, to conduct our analysis and searched all news stories available in the database that met our inclusion criteria over a 16-year period (1997–2012). Second, we developed a search strategy that enabled us to retrieve a large number of news stories using terms and concepts related to PHC rather than restricting our search to only the term ‘primary health care’ and we extended our search to both the titles and full texts of news stories in every section of all newspapers instead of restricting our search to the titles or select sections of the news stories. Third, we read the full text of all the news stories that were retrieved in order to determine their eligibility for inclusion rather than determining eligibility based on only the titles of the news stories. Finally, we used a three-phase strategy that enabled a systematic analysis of the data and two independent raters to code the news stories in the three phases of the analyses in order to improve scientific rigour.

Despite the strengths of the study, a number of limitations should be noted. We limited our research to the print media, whereas other types of media (e.g. television, radio and social media) could also play a role in reporting issues related to PHC. Further, we restricted our analysis to newspaper editions published for at least one full year in LexisNexis and did not include other newspaper editions that were not in this database during the time period covered by the study. Finally, our analysis covered only newspapers published in English language and excluded those published in local South African languages, which may be an important source of information for different language groups in South Africa.

### Findings in relation to other studies

To our knowledge, this is the first study to examine print media coverage of PHC and related research evidence in South Africa. Our finding of an increase in the total number of news stories that mentioned PHC from 2006–2012 (with a peak in 2011) is consistent with the literature documenting a significant growth in the number of, and state support for, non-profit organizations providing home- and community-based care, as well as increased funding of vertical healthcare initiatives by international funders [13,15,17,19,39]. Such vertical healthcare initi atives include HIV prevention and treatment (including antiretroviral therapy) and home-based care, both of which have PHC dimensions.

In addition, the print media coverage of issues relating to CHWs was predominantly about issues relating to remuneration, which is stipulated by the CHW policy frameworks developed by the government in 2003 and 2009, and by the Expanded Public Works Programme, which launched a first phase in 2004 and a second phase in 2009 [15,18,39,42]. Following these developments, there was an increase in funding and other technical support for community-based organizations and CHW stipends and salaries from government and international funding agencies [15,18,19]. However, a number of challenges with the implementation of the CHW policies and the Expanded Public Works Programme have generated an outcry among CHWs and intense debates among researchers, policymakers and stakeholders [15,18,19,39,42-44] which have received a lot of media coverage.

The finding that issues relating to CHWs received the highest level of news coverage is consistent with research that highlights the key role played by CHWs in the delivery of PHC services [15,17,39,44,45]. Our finding about the high level of coverage of HIV/AIDS/TB issues in the news media reflects the high prevalence of HIV/AIDS/TB and various initiatives addressing these diseases and related issues [46,47].

On the other hand, the limited coverage of issues relating to rape and sexual violence in news stories does not reflect the high incidence of rape in South Africa. In 2009, 68,332 cases of rape were reported to the South African Police [48] but this only represents a fraction of rape cases since only 1 in 25 cases of rape are reported to the police [49]. A recent national household survey revealed that 28% of men had raped or sexually assaulted women at least once in their lifetime and 54% of these men had done so on multiple occasions [50]. It is also important to note that the PHC package comprises a range of services to address rape and sexual violence, including treatment and counselling, post-exposure prophylaxis, emergency contraception, support for accessing the criminal justice system, and referral to appropriate health facilities for continuity of care [51].

In addition, there was a limited coverage of health research evidence related to PHC, which is central to evidence-informed health systems [31-33] and in particular there was no news story that discussed systematic reviews. Yet, systematic reviews are considered a type of publication that is well suited to use in supporting evidence-informed health systems [31,33].

### Implications for policy

Our findings reveal the characteristics of news stories that cover issues relating to PHC and illustrate the value of systematically studying patterns in media coverage. For example, some newspapers provided more coverage of issues relating to PHC than others, while further still, other newspapers – notably community newspapers – did not publish any news story that focused on PHC. Given that community newspapers normally cover news from the grassroots, it is surprising that none covered any story on PHC. This finding suggests that community newspapers present untapped opportunities for the dissemination of issues relating to PHC that advocates, stakeholders and policymakers should consider exploring.

By documenting health issues that are receiving either prominent or limited media coverage, our analysis helps highlight issues that are of concern or perhaps should be of concern to policymakers, stakeholders and reporters. The gatekeeping literature suggests that news media coverage does not necessarily reflect the salience of issues in the real world, but that media organizations prioritize items for publication based on what they consider newsworthy [35-37]. For example, with respect to health system arrangements, our findings show that issues relating to accountability of the state sector’s role in financing and delivery of PHC services received the most coverage in the print media during the 16-year study period. While many of the news stories discussed complaints from stakeholders about the delivery of PHC services, others covered announcements from policymakers about policy options and implementation considerations to address the policy problems. A qualitative analysis of the findings could help illuminate this issue but is beyond the scope of the present study.

The fact that there was limited coverage of rape despite its high incidence and the fact that the PHC package in South Africa includes comprehensive care for rape and sexual assault survivors point to an important role for advocates, stakeholders and policymakers. These groups could actively engage the media in the dissemination of important information relating to particular health issues, thereby helping to promote coverage of health issues that is reflective of their salience in the real world. To play this role, they will need to track media coverage of particular health issues in real time.

### Implications for research

Our finding that some issues, such as accountability of the state sector’s role in financing and delivery, received prominent coverage in the print media, while others, such as rape, received limited coverage is consistent with the literature on gatekeeping [35-37]. It could also be related to the skills of the researchers and/or the reporters – it could be that researchers are not communicating their research (making their research findings accessible) to the media or the public or that journalists do not have the required skills and resources to access research evidence [31]. Another possibility is that journalists and media organizations may not consider research evidence newsworthy. Together, these findings suggest an opportunity for research that explores factors facilitating and impeding the coverage of particular issues relating to PHC in the South African media.

We are currently working on a study that employs a qualitative approach to gain a deeper, and more nuanced, understanding of media coverage of issues related to PHC. In addition, we are working on a protocol that investigates factors influencing the news media reporting and researchers’ dissemination of health research findings in South Africa.

## Conclusion

Our study underscores the potential role of media analyses in illuminating patterns in print media coverage of health issues. It provides an understanding of health issues that are prominent in the news media as well as gaps in coverage of specific health issues. Our findings show that policymaking about and implementation of current reforms (e.g. the PHC re-engineering initiative) in South Africa could benefit from a thorough review and understanding of patterns of media coverage of issues about PHC and related research evidence. Our study also suggests that by highlighting patterns in media coverage of research evidence, print media analyses could play a role in improving the climate for evidence-informed health policymaking in the LMIC context.

The findings raise questions about media management agendas and the gatekeeping practices of print media organizations in South Africa and how this might influence coverage of health news. Further, they highlight the need for researchers in LMICs to be more proactive in making use of media analyses and in disseminating their research to the media to help illuminate health-related issues that require the attention of health policymakers, stakeholders and reporters. Information from media analyses could also be useful for providing an indication of potential areas of research for health researchers to explore.

## Additional files

Additional file 1:
**Number of news stories retrieved from LexisNexis using various search terms.** (DOCX 20 kb) Additional file 2:
**Criteria for coding articles.** (DOCX 20 kb) Additional file 3:
**Foci of news stories that mention primary healthcare-related terms.** (DOCX 65 kb)

## Abbreviations

CHW: Community health worker
LMIC: Low- and middle-income countries
NHI: National Health Insurance
PHC: Primary healthcare

## References

[1] World Health Organization. United Nations Children’s Fund. Report of the International Conference on Primary Health Care. Alma-Ata; 1978. http://www.who.int/publications/almaata_declaration_en.pdf.

[2] Walt G (1988). CHWs: are national programmes in crisis?. Health Policy Planning.

[3] Walt G (1990). Community health workers in national programmes: just another pair of hands?.

[4] Narasimhan V, Brown H, Pablos-Mendez A, Adams O, Dussault G, Elzinga G (2004). Responding to the global human resources crisis. Lancet.

[5] Schneider H, Hlophe H, Van Rensburg D (2008). Community health workers and the response to HIV/AIDS in South Africa: tensions and prospects. Health Policy Planning.

[6] Akintola O (2008). Unpaid HIV/AIDS care in southern Africa: forms, context and implications. Fem Econ.

[7] World Health Organization (2002). Community home-based care in resource-limited settings: a framework for action.

[8] World Health Organization. Treat, train, retain. Task shifting: global recommendations and guidelines. Geneva: WHO; 2008. http://www.who.int/healthsystems/task_shifting/en/.

[9] Callaghan M, Ford N, Schneider H (2010). A systematic review of task-shifting for HIV treatment and care in Africa. Hum Resour Health.

[10] Rhode J, Cousens S, Chopra M, Tangcharoensathien V, Black R, Bhutta Z (2008). 30 years after Alma-Ata: has primary health care worked in countries?. Lancet.

[11] Schaay N, Sanders D, Barron P, Roma-Reardon J (2008). International perspective on primary health care over the past 30 years. South African Health Review.

[12] Lewin SA, Munabi-Babigumira S, Glenton C, Daniels K, Bosch-Capblanch X, Van Wyk BE (2010). Lay health workers in primary and community health care for maternal and child health and the management of infectious diseases. Cochrane Database Syst Rev.

[13] Mwai GW, Mburu G, Torpey K, Frost P, Ford N, Seeley J (2013). Role and outcomes of community health workers in HIV care in sub-Saharan Africa: a systematic review. J Int AIDS Soc.

[14] Friedman I, Ijumba P, Padarath A (2005). Community health workers and community caregivers: towards a model of unified practice. South African Health Review.

[15] Schneider H, Lehmann U (2010). Lay health workers and HIV programmes: implications for health systems. AIDS Care.

[16] Russel H, Schneider M (2000). A rapid appraisal of community–based HIV/AIDS care and support programmes in South Africa.

[17] Department of Health (2001). National guideline on home-based care and community-based care.

[18] Akintola O (2015). Public works programme public works programme and primary health care in South Africa: creating jobs for health systems strengthening?. Dev South Afr.

[19] Naledi T, Barron P, Schneider H, Padarath P, English R (2011). Primary health care in SA since 1994 and implications for the new vision on PHC re-engineering. South African Health Review.

[20] Department of Health (2010). Re-engineering Primary Health Care in South Africa. National Department of Health Discussion Paper.

[21] Department of Health (2011). National Health Insurance in South Africa: Policy Paper. Government Notice 657 of 12^th^ August 2011, Gazette Number 34523.

[22] Rabinowitz A (2010). Media framing and political advertising in the patients’ bill of rights debate. J Health Polit Policy Law.

[23] Daw JR, Morgan SG, Thomson PA, Law MR (2013). Here today gone tomorrow: the issue attention cycle and national print media coverage of prescription drug financing in Canada. Health Policy.

[24] Soroka SN (2002). Agenda-setting dynamics in Canada.

[25] Collins PA, Abelson J, Pyman H, Lavis JNL (2006). Are we expecting too much from print media? An analysis of newspaper coverage of the 2002 Canadian healthcare reform debate. Soc Sci Med.

[26] Waddell C, Lomas J, Lavis JN, Abelson J, Shepard CA, Bird-Gayson T (2005). Joining the conversation: newspaper journalists’ views on working with researchers. Healthcare Policy.

[27] Kingdon JW (1995). Agendas, alternatives and public policies.

[28] Barry CL, Jarlenski M, Grob R, Schlesinger M, Gollus SE (2011). News media framing of childhood obesity in the United States from 2000 to 2009. Pediatrics.

[29] Hayes M, Ross IE, Gasher M, Gustein D, Dunn JR, Hackett RA (2007). Telling stories: news media, health literacy and public policy in Canada. Soc Sci Med.

[30] Phillips DP, Kanter EJ, Bednarczyk B, Tastad P (1991). Importance of the lay press in the transmission of medical knowledge to the scientific community. N Engl J Med.

[31] Lavis JN, Lomas J, Hamid M, Sewakambo K (2006). Assessing country-level efforts to link research to action. Bull World Health Organ.

[32] Oxman A, Lavis JN, Lewin S, Fretheim A (2009). SUPPORT Tools for evidence informed health policy-making (STP)1: what is evidence-informed policy making?. Health Res Policy Syst.

[33] Lavis JN (2009). How can we support the use of systematic reviews in policymaking?. PLoS Med.

[34] Cheung A, Lavis JN, Hamandi A, El-Jardali F, Sachs J, Sewakambo N (2011). Climate for evidence-informed health systems: a print media analysis in 44 low- and middle- income countries that host knowledge-translation platforms. Health Res Policy Syst.

[35] Shoemaker PJ, Eichholz M, Kim E, Wrigley B (2001). Individual and routine forces in gatekeeping. Journal Mass Commun Q.

[36] Soroka SN (2012). The gatekeeping function: distributions of information in the media and the real world. J Politics.

[37] Lewin K (1950). Field theory in social science: selected theoretical papers.

[38] Mountcastle-Shah E, Tambor E, Bernhardt BA, Geller G, Karaliukas R, Rodgers JE (2003). Assessing mass media reporting of disease-related genetic discoveries: development of an instrument and initial findings. Sci Commun.

[39] Department of Health (2009). Community care worker policy management framework.

[40] McMaster Health Forum (2013). Health Systems Evidence.

[41] Moat KA, Lavis JNL (2013). 10 best resources for …evidence-informed health policy making. Health Policy Planning.

[42] EPWP (Expanded Public Works Programme). Environment and culture sector EPWP phase ii logframe: 2010–2014. www.epwp.gov.za/documents/Sector%20Documents/Environment%20and%20Culture/Logframe_for_the_EC_Sector_FINAL(2010–2014).pdf. Accessed 5 August 2011.

[43] Ogunmefun C, Friedman I, Mothibe N, Mbatha T (2012). A national audit of home and community-based care (HCBC) organisations in South Africa. Vulnerable Child Youth Studies.

[44] Akintola O, Gwelo NB, Labonte R, Appadu T. The global financial crisis: experiences of and implications for community-based organizations providing health and social services in South Africa. Critical Public Health. 2015. Ahead of print. doi:10.1080/09581596.2015.1085959.

[45] Akintola O (2011). What motivates people to volunteer? The case of volunteer AIDS caregivers in faith-based organizations in KwaZulu-Natal, South Africa. Health Policy Planning.

[46] Joint United Nations Programme on HIV/AIDS (UNAIDS) (2012). Report of the global AIDS epidemic.

[47] World Health Organization (2012). Global Tuberculosis Report.

[48] Naidoo K (2013). Rape in South Africa – a call to action. S Afr Med J.

[49] Gender Links & Medical Research Council (2011). The war @ home: preliminary findings of the Gauteng Gender Violence Prevalence Study.

[50] Jewkes R, Sikweyiya R, Morell R, Dunkle K (2011). Gender inequitable masculinity and sexual entitlement in rape perpetration South Africa: findings of a cross-sectional study. PLoS ONE.

[51] Department of Health (2000). The primary health care package for South Africa – a set of norms and standards.

