# Supplementary material for: Print media coverage of primary healthcare and related research evidence in South Africa
Source: Health Res Policy Syst. 2015 Nov 12;13:68. doi: 10.1186/s12961-015-0051-6 (PMC4643501; doi:10.1186/s12961-015-0051-6)
Supplement: Additional file 1: — Number of news stories retrieved from LexisNexis using various search terms. (DOCX 20 kb) [file 12961_2015_51_MOESM1_ESM.docx]

**Additional file 1 Number of news stories retrieved from LexisNexis using various search terms (N=2504)**

| **Search terms** | **1997** | **1998** | **1999** | **2000** | **2001** | **2002** | **2003** | **2004** | **2005** | **2006** | **2007** | **2008** | **2009** | **2010** | **2011** | **2012** |
| --- | --- | --- | --- | --- | --- | --- | --- | --- | --- | --- | --- | --- | --- | --- | --- | --- |
| Community(-)based care | 0 | 0 | 0 | 0 | 0 | 1 | 2 | 1 | 0 | 2 | 11 | 6 | 15 | 12 | 9 | 4 |
| Community(-)based health care | 0 | 0 | 0 | 0 | 0 | 0 | 0 | 0 | 0 | 0 | 0 | 1 | 0 | 1 | 0 | 4 |
| Community(-)based healthcare | 0 | 0 | 0 | 0 | 0 | 0 | 0 | 0 | 0 | 0 | 1 | 0 | 1 | 0 | 0 | 0 |
| Community(-)based health worker(s) | 0 | 0 | 0 | 0 | 0 | 0 | 0 | 0 | 0 | 0 | 2 | 1 | 0 | 2 | 1 | 0 |
| Community caregiver(s) | 0 | 0 | 0 | 0 | 0 | 0 | 0 | 0 | 0 | 0 | 1 | 2 | 1 | 5 | 3 | 5 |
| Community care worker(s) | 0 | 0 | 0 | 0 | 0 | 0 | 0 | 0 | 0 | 0 | 8 | 1 | 0 | 11 | 4 | 9 |
| Community health worker(s) | 0 | 0 | 0 | 0 | 0 | 1 | 2 | 2 | 3 | 7 | 18 | 16 | 26 | 57 | 48 | 53 |
| Home based care | 0 | 0 | 0 | 0 | 0 | 5 | 4 | 5 | 5 | 11 | 55 | 69 | 90 | 61 | 84 | 81 |
| Home based caregiver(s) | 0 | 0 | 0 | 0 | 0 | 1 | 0 | 0 | 0 | 0 | 2 | 1 | 12 | 10 | 1 | 3 |
| Home based carer(s) | 0 | 0 | 0 | 0 | 0 | 1 | 0 | 0 | 0 | 0 | 2 | 8 | 4 | 6 | 4 | 5 |
| Lay health worker(s) | 0 | 0 | 0 | 0 | 0 | 0 | 0 | 0 | 0 | 0 | 0 | 0 | 0 | 2 | 0 | 0 |
| Primary health care | 0 | 1 | 0 | 2 | 0 | 2 | 4 | 3 | 2 | 20 | 79 | 77 | 147 | 164 | 229 | 176 |
| Primary healthcare | 0 | 0 | 0 | 0 | 0 | 2 | 12 | 9 | 3 | 6 | 57 | 71 | 88 | 131 | 176 | 137 |
| Volunteer caregiver(s) | 0 | 0 | 0 | 0 | 0 | 1 | 0 | 0 | 0 | 0 | 3 | 1 | 2 | 1 | 2 | 1 |
| Volunteer carer(s) | 0 | 0 | 0 | 0 | 0 | 1 | 0 | 0 | 0 | 0 | 0 | 0 | 0 | 0 | 2 | 0 |
| **Total** | 0 | 1 | 0 | 2 | 0 | 15 | 24 | 20 | 13 | 46 | 239 | 254 | 386 | 463 | 563 | 478 |
