# Supplementary material for: Print media coverage of primary healthcare and related research evidence in South Africa
Source: Health Res Policy Syst. 2015 Nov 12;13:68. doi: 10.1186/s12961-015-0051-6 (PMC4643501; doi:10.1186/s12961-015-0051-6)
Supplement: Additional file 2: — Criteria for coding articles. (DOCX 20 kb) [file 12961_2015_51_MOESM2_ESM.docx]

Additional file 2 Criteria for coding articles

Primary focus

A primary health care related term/concept - primary health care, community care and home care - should be in the heading/topic of the news story or the first paragraph. A PHC related term could also be the subject of discussion leading up to the use of the term in the first few sentences. Some articles start with a few sentences that then lead up to the use of the PHC related term. The key issue for the rater is to determine whether or not the main focus of the article is PHC.

Secondary focus

A primary health care term/concept is not in the heading/topic or first paragraph of the article and the news story does not have a focus on any of the PHC related terms. It could be that PHC related term is mentioned more than twice or in a sentence but the raters need to determine whether primary health care is the focus of the article or whether the article just made a reference to the term while discussing other issues.

Mention

One or two unrelated sentences or references to PHC related term/concept and not enough discussion to warrant classification as secondary focus. Most of the articles that fall into this category should make a passing reference to a PHC related term.
