# Supplementary material for: Print media coverage of primary healthcare and related research evidence in South Africa
Source: Health Res Policy Syst. 2015 Nov 12;13:68. doi: 10.1186/s12961-015-0051-6 (PMC4643501; doi:10.1186/s12961-015-0051-6)
Supplement: Supplementary file 3 [file 12961_2015_51_MOESM3_ESM.docx]

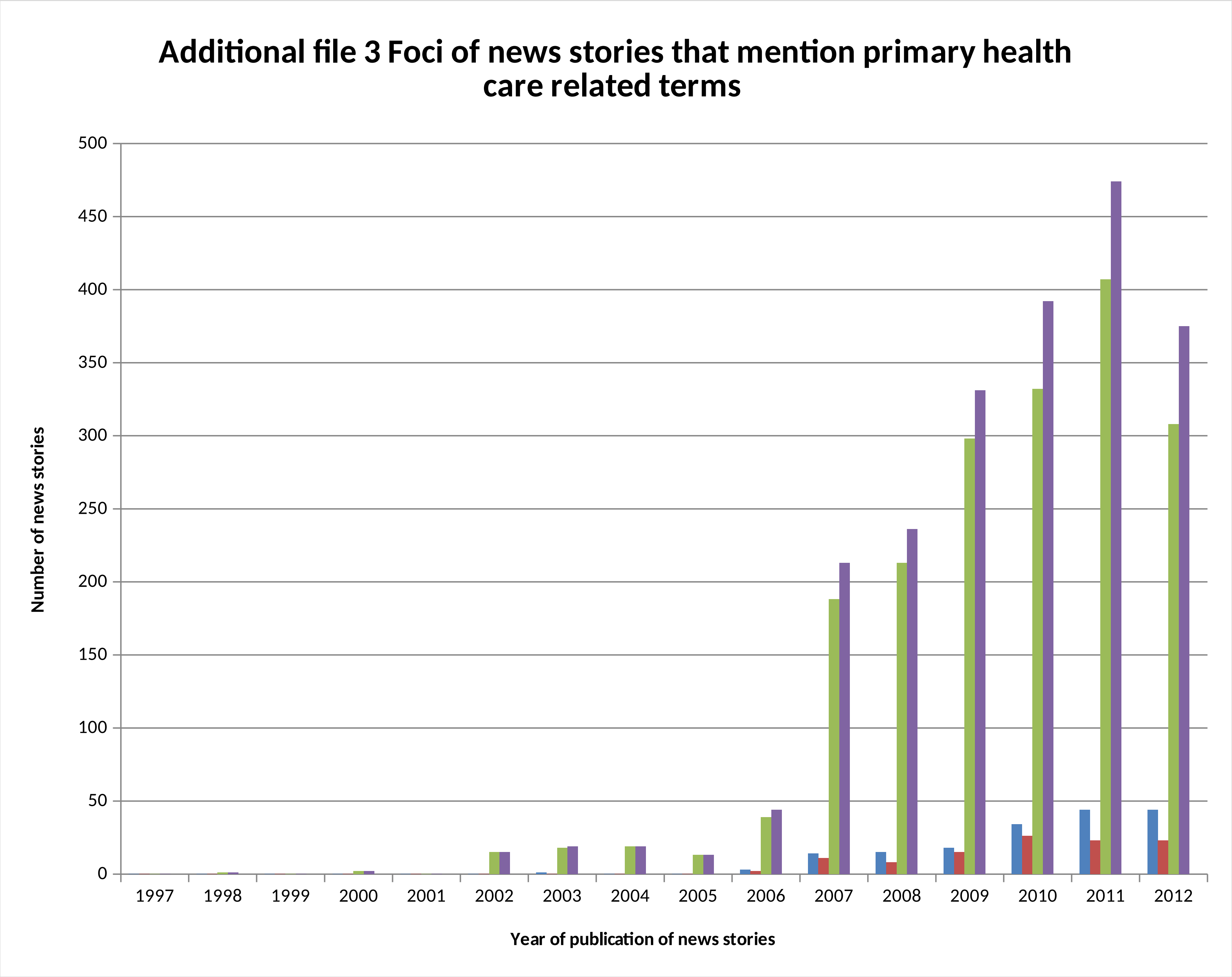
Additional file 3 Foci of news stories that mention primary health care related terms
Number of news stories
Year of publication of news stories
0
50
100
150
200
250
300
350
400
450
500
1997
1998
1999
2000
2001
2002
2003
2004
2005
2006
2007
2008
2009
2010
2011
2012
